# Inactivation of *Minar2* in mice hyperactivates mTOR signaling and results in obesity

**DOI:** 10.1016/j.molmet.2023.101744

**Published:** 2023-05-26

**Authors:** Saran Lotfollahzadeh, Chaoshuang Xia, Razie Amraei, Ning Hua, Konstantin V. Kandror, Stephen R. Farmer, Wenyi Wei, Catherine E. Costello, Vipul Chitalia, Nader Rahimi

**Affiliations:** 1 Renal Section, Department of Medicine, Boston University Chobanian & Avedisian School of Medicine, Boston, MA, USA; 2 Center for Biomedical Mass Spectrometry, Boston University Chobanian & Avedisian School of Medicine, Boston, MA, USA; 3 Department of Pathology and Laboratory Medicine, Boston University Chobanian & Avedisian School of Medicine, Boston, MA, USA; 4 Biomed Research Center, Boston University Chobanian & Avedisian School of Medicine, Boston, MA, USA; 5 Department of Biochemistry, Boston University Chobanian & Avedisian School of Medicine, Boston, MA, USA; 6 Department of Pathology, Beth Israel Deaconess Medical Center, Harvard Medical School, Boston, MA, 02215, USA; 7 Veterans Affairs Boston Healthcare System, Boston, MA, USA; 8 Institute of Medical Engineering and Sciences, Massachusetts Institute of Technology, Cambridge, MA, USA

**Keywords:** Minar2, mTOR, mTORC1, Raptor, Obesity, Diabetes

## Abstract

**Objective:**

Obesity is a complex disorder and is linked to chronic diseases such as type 2 diabetes. Major intrinsically disordered NOTCH2-associated receptor2 (MINAR2) is an understudied protein with an unknown role in obesity and metabolism. The purpose of this study was to determine the impact of Minar2 on adipose tissues and obesity.

**Method:**

We generated Minar2 knockout (KO) mice and used various molecular, proteomic, biochemical, histopathology, and cell culture studies to determine the pathophysiological role of Minar2 in adipocytes.

**Results:**

We demonstrated that the inactivation of Minar2 results in increased body fat with hypertrophic adipocytes. Minar2 KO mice on a high-fat diet develop obesity and impaired glucose tolerance and metabolism. Mechanistically, Minar2 interacts with Raptor, a specific and essential component of mammalian TOR complex 1 (mTORC1) and inhibits mTOR activation. mTOR is hyperactivated in the adipocytes deficient for Minar2 and over-expression of Minar2 in HEK-293 cells inhibited mTOR activation and phosphorylation of mTORC1 substrates, including S6 kinase, and 4E-BP1.

**Conclusion:**

Our findings identified Minar2 as a novel physiological negative regulator of mTORC1 with a key role in obesity and metabolic disorders. Impaired expression or activation of MINAR2 could lead to obesity and obesity-associated diseases.

## Highlights

• Inactivation of Minar2 in mice predisposes mice to diet-induced obesity.
• Minar2 KO mice on high-fat diet develop obesity and display impaired glucose metabolism.
• Minar2 interacts with Raptor and regulates mTORC1 signaling.

## Graphical abstract

**Figure:**
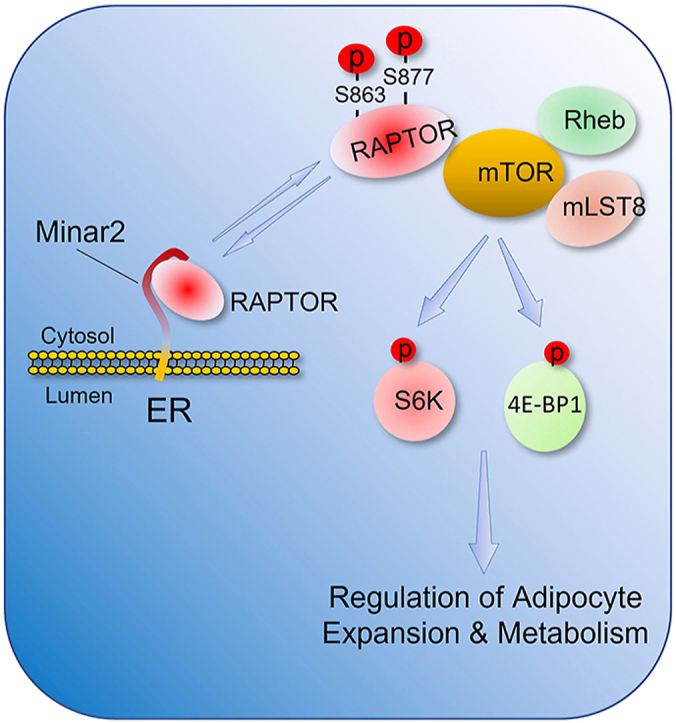

## 1 Introduction

Obesity is a complex disorder that develops from a chronic imbalance between energy intake and energy expenditure and is linked to the development of diseases such as type 2 diabetes [4]. Obesity and obesity-related conditions are also associated with the severity of COVID-19 [1,44]. The mechanistic target of rapamycin (mTOR) signaling plays a central role in the regulation of growth, adipogenesis, and metabolism [5,14,54]. mTOR is a conserved serine/threonine protein kinase and can exist in two functionally and structurally distinct multiprotein complexes; mTOR complex 1 (mTORC1) and mTOR complex 2 (mTORC2). mTORC1 contains mTOR, Raptor, mLST8 (mammalian lethal with Sec-13 protein 8), and others, whereas mTORC2 contains mTOR, Deptor, mLST8, Rictor (rapamycin-insensitive companion of mTOR) and others [20,40]. Raptor is a specific and essential positive regulator of mTORC1 [25]. While full-body knockout of Raptor in mice is embryonically lethal [14], targeting Raptor in mature adipocytes via the adiponectin-Cre system demonstrated that raptor^Adipoq-cre^ mice were resistant to high fat-diet-induced obesity and adipose tissue expansion leading to lipodystrophy [28].

Major intrinsically disordered NOTCH2-associated receptor 1 & 2 (MINAR1 and MINAR2) were first identified in our laboratory [17,18]. MINAR1 and MINAR2 each are composed of an extracellular domain, and a single transmembrane domain with a short cytoplasmic tail. Human MINAR2 is located on the chromosome 5q23.3 and encodes a 190 amino acid protein [17]. While MINAR1 is present in the cytoplasm and plasma membrane [18], MINAR2 is prominently detected in the endoplasmic reticulum (ER) compartments [17]. MINAR1 and MINAR2 function as negative regulators of cell proliferation [17,18,57]. A recent study suggested that MINAR1 could inhibit cell proliferation, in part, via regulating mTOR activity [57]; however, the role and mechanism of MINAR2 in mTOR activation are not known. In this study, we demonstrate that the whole-body inactivation of *Minar2* in mice increases adipose tissue and results in mTOR hyperactivation. Minar2 is a physiological negative regulator of mTORC1 with a major role in obesity.

## 2 Results

### 2.1 Minar2 is expressed in mouse and human adipose cells

**Figure 1:**
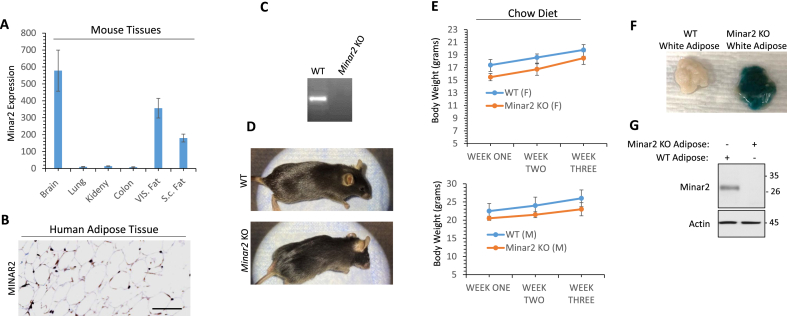
**Expression of Minar2 in adipocytes and generation of *Minar2* knockout mice**: (**A**) Minar2 mRNA expression in mouse adipose tissues. (**B**) MINAR2 protein expression in human adipose tissue. Image magnification 100 μM. (**C**) Genotyping of *Minar2* KO mice. (**D**) Image of three months old *Minar2* KO and control wild-type (WT) littermate mice. (**E**) The growth rate of *Minar2* KO and control WT mice on a chow diet (n = 5/group). (**F**) Lac-Z staining of adipose tissues from *Minar2* and WT mice. (**G**) Western blot analysis of adipose tissues from *Minar2* KO and WT mice.

To investigate the role of Minar2 in obesity, we examined the expression of Minar2 in the adipose tissue of mice feed on chow diet. Quantitative polymerase chain reaction (qPCR) analysis showed that Minar2 is expressed in mouse adipose tissues. Minar2 mRNA levels were particularly higher in the visceral adipose compared to the subcutaneous adipose tissue (Figure 1A). As previously reported, Minar2 is highly expressed in the various brain compartments [17] and is here used as a positive control. Expression of Minar2 in the lung, kidneys, and colon was very low/negligible (Figure 1A). Immunohistochemistry (IHC) staining of human adipose tissue further showed that MINAR2 is also expressed in human adipose tissue (Figure 1B). In addition, we analyzed multiple RNA-seq datasets derived from the previous analysis on adipocytes via Gene Expression Omnibus “GEO profile". A study showed that MINAR2 is expressed in the visceral fat [12]. A similar study also showed that MINAR2 is expressed in the subcutaneous and visceral adipose tissue in lean and obese pre-pubertal children [48]. MINAR2 also is expressed in CD34+ white adipocytes from the breast lipotransfer aspirates [29] and in the CD14+ visceral adipose tissue [8]. Moreover, MINAR2 is expressed in adipose stem cells (ASC) from the subcutaneous white adipose tissue [35] and in the progenitor cells of neck and subcutaneous adipose [49].

To study the role of MINAR2 in obesity, we examined a recently developed whole-body homozygous *Minar2* knockout (KO) mouse where the exon two of *Minar2* was replaced with the *lacZ* gene [19]. Genetic phenotyping of *Minar2* KO mice confirmed the inactivation of *Minar2* (Figure 1C). *Minar2* deficiency is not embryonically lethal, and *Minar2* KO mice are fertile. However, *Minar2* KO mice appeared to be slightly smaller (Figure 1D), though their reduced bodyweights compared to the wild-type (WT) littermates on normal chow diet were not statistically significant (Figure 1E). X-gal staining of the adipose tissue (Figure 1F) and western blotting (Figure 1G) of whole cell lysates from the adipose tissue of *Minar2* KO mouse confirmed the loss of *Minar2* in the adipose tissue.

### 2.2 Inactivation of *Minar2* increases body fat mass in a non-high fat diet

**Figure 2:**
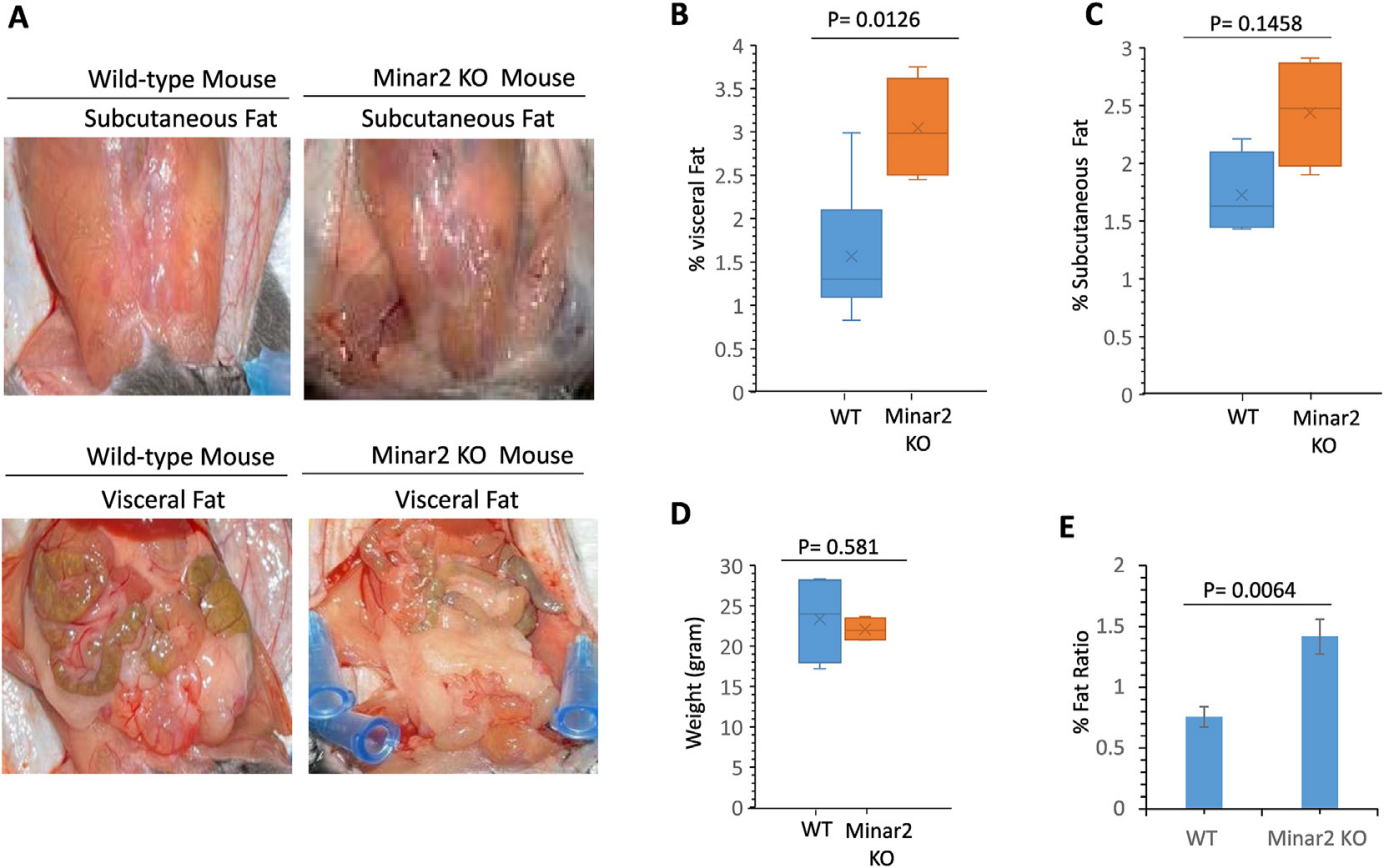
**Inactivation of *Minar2* increases adipose fat ratio in mice on a chow diet**. (**A**) Representative images of fat accumulation in *Minar2* KO and WT mice (6 weeks old). (**B, C**) % visceral and subcutaneous fat mass of *Minar2* KO and control WT mice (n = 5/group, 6 weeks old). (**D**) Total body weight of *Minar2* KO and WT mice (n = 5, 6 weeks old). (**E**) Fat ratio of *Minar2* KO and WT mice (n = 5/group, 6 weeks old).

Having determined that Minar2 is expressed in fat cells, we examined whether the loss of *Minar2* affects body fat mass. We analyzed *Minar2* KO mice adipose mass via magnetic resonance imaging (MRI). MRI analysis revealed that the adipose mass of *Minar2* KO mice on a chow diet was three-fold higher than that of the age- and sex-matched control WT littermates (S. Figure 1A). The average adipose mass of *Minar2* KO mice was 6.8% versus 2.6% in the WT mice (S. Figure 1B). The representative gross analysis of *Minar2* KO and WT mice is shown (Figure 2A). Further analysis showed that the visceral and subcutaneous fat mass of *Minar2* KO mice was significantly higher compared to the control WT mice (Figure 2B and C).

Curiously, despite their relatively smaller size (Figure 2D), the fat ratio of *Minar2* KO mice compared to their total body weights was markedly higher (13.7% fat compared to 6.6% in WT mice, P = 0.0064) (Figure 2E). Altogether, these data indicate that the inactivation of *Minar2* in mice increases fat cell mass, which could lead to obesity.

### 2.3 *Minar2* KO mice are prone to high-fat diet-induced obesity

**Figure 3:**
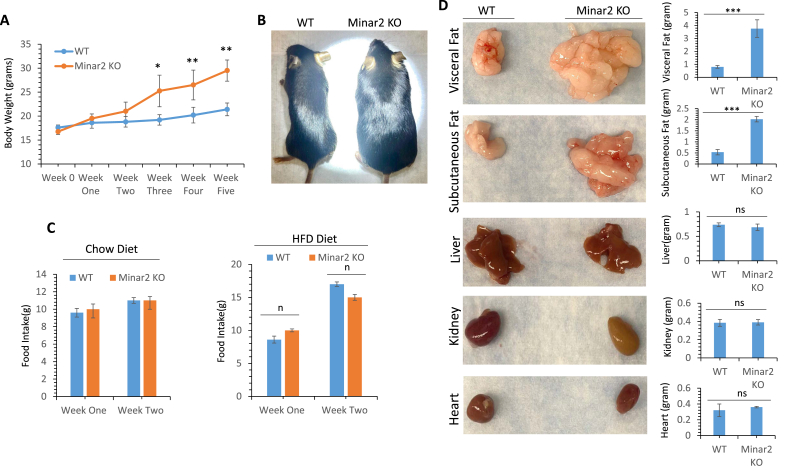
***Minar2* KO mice are prone to diet-induced obesity**. (**A**) Weight gain of *Minar2 KO* and WT mice (6 weeks old, n = 5) on a high-fat diet (HFD). (**B**) Representative images of *Minar2* KO and WT mice after six weeks on HFD. (**C**) Food consummation of *Minar2* KO and WT mice on a chow diet or HFD (n = 5/group). (**D**) Representative fat tissues and other organs of *Minar2* KO and WT mice after six weeks on HFD.

To investigate whether *Minar2* KO mice are susceptible to diet-induced obesity, we challenged *Minar2* KO and littermate control WT mice with a high-fat diet (HFD) and monitored their weight gain and food consumption over five weeks. The result showed that *Minar2* KO mice gained weight more rapidly than WT mice (Figure 3A,B). After five weeks on the HFD, *Minar2* KO mice had gained 77.3% weight over their starting weight, whereas WT mice gained only 21.6%. *Minar2* KO mice in the fifth week on HFD were 37.85% heavier than WT mice (Figure 3A). To examine the cause of the weight gain in *Minar2* KO mice, we first examined whether the food intake of *Minar2* KO mice is higher than WT mice. The food consumption of *Minar2* KO mice was not noticeably different from WT mice on both chow and HFD diets (Figure 3C), indicating that the observed weight gain in *Minar2* KO mice is not associated with increased food consumption. Next, we asked whether the increase in weight of *Minar2* KO mice is linked to the enlargement of organs or fat accumulation. We measured weights of visceral and subcutaneous fat depots and other major organs. The visceral and subcutaneous fat depots weighed significantly more in *Minar2* KO than in WT mice (Figure 3D). However, there were no significant differences in weights of the liver, kidneys, or heart of *Minar2* KO and WT mice (Figure 3D), indicating that the effect of the inactivation of *Minar2* was specific to adipose tissue. This observation further suggests that the inactivation of *Minar2* in mice confers susceptibility to diet-induced obesity.

### 2.4 Inactivation of *Minar2* induces adipocyte hypertrophy and impaired glucose tolerance

**Figure 4:**
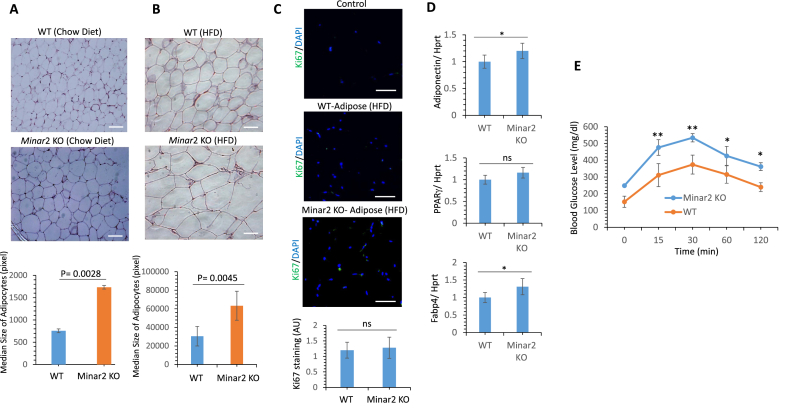
**Inactivation of *Minar2* in mice induces hypertrophy in adipocytes**. (**A**) H&E staining of adipocytes harvested from *Minar2* KO or WT mice on chow diet. Scale bars, 50 µm (**B**) H&E staining showing adipocytes harvested from *Minar2* KO or WT mice on high-fat diet (HFD). Scale bars, 50 µm. (**C**) Ki67 staining of adipose tissues harvested from *Minar2* KO or WT mice. Scale bars, 50 µm. (**D**) Glucose tolerance test on *Minar2* KO and WT mice on HFD. Mice were fasted for overnight before administration of glucose (7 mice/group, 6 weeks old).

Adipocyte hypertrophy (increase in adipocyte cell size) is the main mechanism for adult fat mass expansion [26,46], where the enlargement of cells facilitates the excess energy intake and is linked to obesity and metabolic disorders, [22,32]. We investigated whether the inactivation of *Minar2* induced hypertrophic adipocytes in mice. H&E staining of visceral adipocytes from *Minar2* KO mice on a chow diet showed that adipocytes were significantly hypertrophic compared to age- and sex-matched WT littermates (Figure 4A). Remarkably, adipocyte cell size further increased in *Minar2* KO mice on HFD (Figure 4B). Furthermore, we counted the adipocyte cell numbers in each fat pat. Although we noted an increase in the adipocyte cell numbers in *Minar2* KO mice, this increase in cell number, however, was not statistically significant (S. Figure 2). Additionally, we also did not observe an increase in cell proliferation in adipocytes of *Minar2* KO mice as determined by Ki67 staining (Figure 4C), indicating that Minar2 mostly regulates hypertrophic but not a hyperplasic proliferation of adipocytes. Next, we examined whether the inactivation of *Minar2* in mice results in the development of more mature adipocytes. We assessed the expression of several known biomarker genes of mature adipocytes, including adiponectin, FABP4, and PPARγ. There was a relatively small increase in the mRNA levels of adiponectin and FABP4, but not PPARγ (Figure 4D). This finding suggests that adipocyte cell expansion but not the maturation of fat cells is largely associated with increased fat cell mass in *Minar2* KO mice.

Given a strong link between the hypertrophy of adipocytes and impaired glucose metabolism [22,32], we performed a glucose tolerance test to determine the ability of *Minar2* KO mice for glucose clearance. To this end, *Minar2* KO and WT mice on HFD were fasted for 12–14 h, followed by an IP injection of glucose (2 g/kg). Blood was collected from the mice and glucose levels were measured using a glucometer before the injection (to determine basal blood glucose level) and then at 15,30, 60, and 120 min after the injection. The baseline glucose level of *Minar2* KO mice was 242 mg/dl (SD = 16.3) compared to 152 mg/dI (SD = 32.2) in WT mice, which was 59.2% higher than in WT mice (Figure 4E), indicating that inactivation of *Minar2* resulted in the impaired glucose metabolism. More importantly, blood glucose levels in *Minar2* KO mice compared to WT mice remained high up to 120 min after IP injection of glucose (Figure 4E), suggesting that glucose metabolism is significantly impaired in *Minar2* KO mice.

### 2.5 Minar2 interacts with proteins that are involved in the metabolism, obesity and mTOR signaling

**Figure 5:**
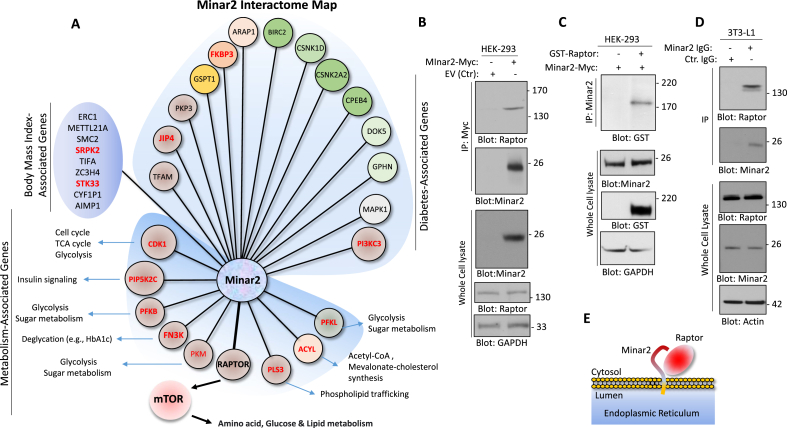
**Identification of raptor as a Minar2 binding protein**. (**A**) The Minar2 interactome map. Minar2 interacting proteins are grouped into three major categories: Proteins involved in the metabolism, body-mass index, or diabetes. (**B**) Western blot analysis showing the binding of Minar2-myc with endogenously expressed raptor in HEK-293 cells. (**C**) Western blot analysis demonstrating the binding of Minar2-myc with the ectopically expressed GST-raptor in HEK-293 cells. (**D**) Western blotting analysis showing the binding of endogenously expressed Minar2 and raptor in 3T3-L1 cells. (**E**) Proposed model of interaction of MINAR2 with raptor.

To understand the mechanisms by which Minar2 elicits its biological activity, we decided to identify the Minar2 interactome. We immunoprecipitated Minar2 from HEK-293 cells expressing Minar2-Myc, and performed liquid chromatography-tandem mass spectrometry (LC-MS/MS) analysis to identify proteins that were pulled down along with Minar2-Myc. Our analysis identified 327 proteins that were selectively detected in the Minar2 group. Analysis of the corresponding genes via the Database for Annotation, Visualization, and Integrated Discovery (DAVID) revealed that 85 of these genes are associated with diabetes, metabolism, or body mass index (data not shown). The most important Minar2-interacting proteins linked to diabetes, metabolism, and body mass index are shown in Figure 5A. The top Minar2-interacting proteins with a link to metabolism were FN3K, raptor, PFKL, ACYL, PLS3, PKM, PFKB, PIP5K2C, and CDK1 (Figure 5A). FN3K (fructosamine-3-kinase) catalyzes the phosphorylation of fructosamines and is responsible for the formation of fructose 3-phosphate (F3P), decomposition of F3P can lead to the formation of 3-deoxyglucosone (3DG) and is known to contribute to diabetic complications [7,9,47] and elevated HbA1c levels in diabetic individuals [30,45]. The regulator-associated protein of mTOR (Raptor) is a specific and essential component of the mammalian TOR complex 1 (mTORC1), which is essential for mTOR kinase activation and substrate recognition [42]. The mTOR pathway is a master nutrient sensor, which plays a key role in obesity and other metabolic disorders such as diabetes [53]. PFKL (ATP-dependent 6-phosphofructokinase), catalyzes the phosphorylation of d-fructose 6-phosphate to fructose 1,6-bisphosphate by ATP, the first step of glycolysis [56]. ACYL/CTE-I (acyl-coenzyme A thioesterase1) catalyzes the hydrolysis of acyl-CoAs into free fatty acids and coenzyme A [13]. A brief description of the involvement of these proteins in metabolism is shown (Figure 5A). We found fourteen Minar2-interacting proteins with an established link to diabetes. Among them was JNK-interacting protein 4 (JIP4). JIP4 is a scaffold protein, and its activity is associated with metabolic stress, insulin resistance, and diabetes [21,51] and mice deficient for JIP4 are resistant to diet-induced obesity [21]. The most important Minar2-binding proteins with a strong link to diabetes are shown (Figure 5A). There were also nine Minar2 interacting proteins with a potential link to body mass-index. For example, SRSF protein kinase 2 (SRPK2), which is a serine/arginine-rich protein-specific kinase that phosphorylates its substrates at serine residues located in regions rich in arginine/serine dipeptides (RS) domains and is involved in the phosphorylation of SR splicing factors and the regulation of splicing and its expression is linked to the body mass index [37]. Similarly, the expression of serine/threonine kinase 33 (STK33) is closely associated with body mass [39].

### 2.6 Minar2 binds to Raptor and inhibits mTORC1 activation

Given the central role of mTOR signaling in metabolism and obesity [6,31], we decided to investigate the functional importance of Minar2 interaction with Raptor and its implication in mTOR signaling. First, we confirmed the binding of Minar2 with Raptor in HEK-293 cells ectopically expressing Minar2 (Figure 5B), or Raptor-FLAG (Figure 5C) via co-immunoprecipitation assays. Similarly, we show that Minar2 interacts with Raptor in 3T3-L1 cells endogenously expressing Minar2 and Raptor (Figure 5D). Proposed model of interaction of Minar2 with Raptor (Figure 5E).

To better understand the interaction of Minar2 with Raptor, we asked whether Raptor co-localizes with Minar2. Our previous studies demonstrated that Minar2 is predominately expressed in the ER. The N-terminus domain of Minar2 interacts with Raptor, which is exposed outside and its short C-terminus resides in the lumen [19]. Staining of HEK-293 cells expressing GST-Raptor showed that Raptor is present in the cytoplasm, and it is also co-localized with Minar2 (S. Figure 3 A and B). In agreement with the previous studies that have shown the mTORC1 complex is trafficked to the lysosome for its full activation [33,41], we show that Raptor also localizes with mCherry-lysosomes-20/LAMP1 (lysosome-associated membrane glycoprotein 1), a lysosomal marker (S. Figure 3C).

**Figure 6:**
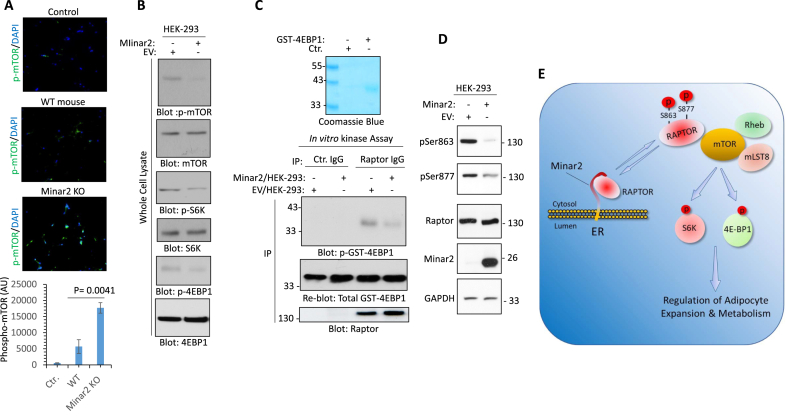
**Minar2 regulates mTORC1 activation and inhibits phosphorylation of raptor**. (**A**) Immunofluorescence staining showing hyperphosphorylation (Ser2448) of mTOR in the adipocytes harvested from *Minar2* KO mice or control WT mice. (**B**) Western blotting analysis showing expression of Minar2 in HEK-293 cells inhibits phosphorylation of mTOR on Ser2448. Cells were starved for 16 h, then stimulated with insulin (100 ng/mL, 30min), cells were lysed and whole cell lysates were subjected to western blot analysis. (**C**) In vitro mTORC1 kinase assay using GST-4EBP1 as a substrate. (**D**) Western blot analysis showing expression of Minar2 in HEK-293 cells inhibits phosphorylation of raptor on Ser863 and Ser877. (**E**) Summary and proposed model for interaction of Minar2 with Raptor. In normal physiological conditions, Minar2 interacts with Raptor and limits its interaction with mTORC1 complex, which leads to temporal regulation of mTORC1 activity. Inactivation of *Minar2* disrupts the temporal regulation of mTORC1, leading to hyperactivation of mTORC1 signaling.

Considering the central role of Raptor in the activation and signaling of mTOR and our evidence for its interaction with Minar2, we investigated the role of Minar2 in mTORC1 signaling. We first asked whether the inactivation of Minar2 affects mTOR phosphorylation. We stained adipose tissues harvested from WT and *Minar2* KO mice with a phospho-mTOR antibody (pSer2448). Phosphorylation of Ser2448 is required for the kinase activation of mTOR [34,36]. Our results showed a significant increase in Ser2448 phosphorylation of mTOR in *Minar2* KO adipocytes (Figure 6A). Next, we tested whether over-expression of Minar2 in HEK-293 cells can affect insulin-mediated phosphorylation of mTOR. Western blot analysis showed that cells expressing Minar2 has markedly reduced mTOR Ser2448 phosphorylation (Figure 6B). Likewise, phosphorylation of the key substrates of mTOR including, S6-kinase and 4EBP-1 was also reduced (Figure 6B). To gain further insight into the mechanism by which Minar2 inhibits phosphorylation of mTOR, we determined the kinase activation of mTOR by measuring the *in vitro* phosphorylation of 4EBP1 as an mTORC1 substrate. The result showed that mTORC1 kinase activity toward 4EBP1 is significantly reduced in HEK-293 cells that over-express Minar2 (Figure 6C).

Raptor is phosphorylated at multiple sites by various kinases including mTOR [3,10]. We asked whether Minar2, in addition to its interaction with Raptor, also affects the phosphorylation of Raptor. Our initial analysis via LC-MS/MS identified Ser863 and Ser877 that are phosphorylated on Raptor in HEK-293 cells (S. Figure 4A, 3B). Next, we examined whether phosphorylation of Raptor at Ser863 and Ser877 is modulated by Minar2. Our data showed that Raptor is phosphorylated on Ser863 and Ser877 in HEK-293 cells expressing control vector (EV), but phosphorylation of these sites is reduced in HEK-293 cells expressing Minar2 (Figure 6D). Taken together, our data suggest that Minar2 interacts with Raptor, and modulates its phosphorylation and interaction with mTORC1 (Figure 6E).

## 3 Discussion

In this study, we demonstrate that the inactivation of *Minar2* in mice results in a dramatic effect on diet-induced obesity. *Minar2* KO mice display hypertrophic adipocytes and impaired glucose tolerance and metabolism. Adipocyte hypertrophy is considered a key mechanism of adult fat mass expansion [26,46], which is linked to obesity and other metabolic disorders [22,32]. Furthermore, we provide evidence that Minar2 interacts with Raptor and inhibits its interaction with mTOR leading to reduced mTOR kinase activity. These results suggest that Minar2 through its interaction with Raptor regulates mTOR activity and plays an important role in adipocyte function and obesity. Unlike *Minar2* KO mice, adipose-specific Raptor knockout mice, which positively regulates mTORC1 activity, results in lean mice that are resistant to diet-induced obesity [28]. On the contrary, 4 E-BP1 and 4 E-BP2 KO mice, which are negatively regulated by mTORC1 activity, result in increased obesity [27]. Moreover, unlike Raptor KO mice, which display lower basal glucose levels and improved glucose clearance [28]. *Minar2 KO* mice have higher basal glucose levels and reduced glucose clearance. However, *Minar2* KO mice exhibit normal food intake, which is similar to Raptor KO mice [28], indicating that Raptor activity is not associated with food intake. This inverse correlation between the observed *Minar2* KO phenotype and the adipose-specific rRaptor KO mice suggests that the key effects of Minar2 could be mediated via its interaction with Raptor and regulation of mTORC1 activity in adipocytes. In agreement with this idea, mTOR is hyperactivated in the adipocytes of *Minar2* KO mice and over-expression of Minar2 in cell culture inhibits mTORC1 kinase activity and phosphorylation of key mTORC1substrates, including S6 kinase and 4EBP1. We found that Raptor is co-localized with the Minar2 in the ER proximity and Minar2 inhibits the interaction of raptor with mTOR, which is essential for mTORC1 activation and substrate phosphorylation. Our results show that Minar2 inhibits the phosphorylation of Ser863 and Ser877 on Raptor, sites which are known to be phosphorylated by mTOR and other kinases such as TKB1 and Cdc2 [3,10,52]. This suggests that Minar2 by recutting Raptor limits the interaction of Raptorwith mTOR and other kinases and hence hinders its phosphorylation.

Aside from Raptor, Minar2 interacts with a range of other proteins, which are associated with diabetes, metabolism, and the body mass-index. However, it remains to be determined how Minar2 interaction with these proteins could influence their function. Our previous studies revealed that loss of Minar2 in mice impairs motor function and results in Parkinson's disease-like symptoms [17]. Emerging evidence now indicates that mTOR and autophagy are critical aspects of the pathogenesis of Parkinson disease [43,55], suggesting that deregulation of Minar2-mediated mTORC1 activity could, in part, also account for the observed impaired motor function in *Minar2* KO mice. Curiously, interfering with mTORC1 signaling in various animal models, including yeast, flies, and worms has also been shown to extend lifespan [23,24,38,50]. However, it remains to be studied whether *Minar2* KO mice may have a shorter lifespan due to the hyperactivation of mTOR. In conclusion, we have found that Minar2 plays a critical role in adipocyte expansion and obesity. Minar2 regulation of mTORC1 via its interaction with Raptor, could be responsible, in part, for the diet-induced obesity observed in *Minar2* KO mice. We suggest that Minar2 is a physiological negative regulator of mTORC1 and could explored for the development of strategies for weight control.

However, despite the clear effect of loss of *Minar2* on fat mass and glucose metabolism in mice, our study has several limitations; First, this is a whole-body knockout, and hence the direct role of loss of *Minar2* in adipocytes needs further investigation, which requires generating an adipocyte-specific and more desirably an inducible animal model system. We previously reported that *Minar2* KO mice when subjected to a particular physical challenge display some form of motor impairment [17]. Although we did not observe an apparent reduced physical activity with *Minar2* KO mice, further examination is needed to fully exclude this possibility. More importantly, given that Minar2 is expressed in various brain compartments [17] it remains to be investigated, whether expression of Minar2 in the brain plays a particular role in the observed phenotype of *Minar2* KO mice in obesity.

## 4 Materials and methods

### 4.1 Cell culture

HEK-293 and 3T3-L1 cells were maintained in DMEM medium containing 10% fetal bovine serum supplemented with penicillin and streptomycin. HEK-293 cells stably expressing Minar2 were transfected with raptor construct or other plasmids as indicated in the figure legends via PEI (polyethylenimine) as described [15]. After 48 h transfection, cells were lysed and subjected to immunoprecipitation or western blotting as described in the figure legends.

### 4.2 Plasmids and antibodies

Construction of Minar2-Myc-pQCXIP and Minar2-emarald (EM)-pLNCX^2^ (accession # NM_001257308.1) were previously described [17]. The full sequence of Minar2-myc and Minar2-emerald is provided (S. Figure 5). pCMV-GST-Raptor, Raptor-FLAG and GST-4E-BP1 constructs were previously described [11].

### 4.3 Immunoprecipitation and western blot analyses

Cells were lysed in CHAPS buffer (40 mM HEPES (pH7.4), 2.5 mM MgCl2 and 0.3% CHAPS) supplemented with protease and phosphatase inhibitors or with EB lysis buffer (10 mM Tris–HCl, 10% glycerol, pH 7.4, 5 mM EDTA, 50 mM NaCl, 50 mM NaF, 1% Triton X-100, 1 mM phenylmethylsulfonyl fluoride, 2 mM Na3VO4, and 20 μg/mL aprotinin). The cell lysates were clarified by centrifugation at 12,000 rpm for 15 min at 4 °C and Equal amounts of whole cell lysates (WCL) were resolved by SDS-PAGE and immunoblotted with the indicated antibodies. Normalized whole-cell lysates were subjected to immunoprecipitation by incubation with appropriate antibodies as shown in the figure legends. Immunocomplexes were captured by incubation with either protein A-Sepharose or protein G Agarose beads. The samples were boiled for 5 min at 95 °C, the immunoprecipitated proteins were subjected to western blot analysis. In some cases, membranes were stripped by incubation in a stripping buffer (6.25 mM Tris–HCl, pH 6.8, 2% SDS, and 100 mM β-mercaptoethanol) at 50 °C for 30 min, washed in Western Rinse buffer (20 mM Tris and 150 mM NaCl), and re-probed with the antibody of interest. The blots were scanned and subsequently quantified using ImageJ (NIH).

### 4.4 Generation and purification of recombinant GST-4EBP1 protein

GST-4EBP1 was purified from the BL21 (DE3) *Escherichia coli* transformed with 4EBP1-pGEX-4T2 construct. A single colony was grown in 5 mL Luria–Bertani (LB) medium overnight at 37 °C. The culture was expanded into 250 mL LB medium until an optical density of 0.4–0.6. The protein expression was induced by 0.1 mM isopropyl-β-D-thiogalactoside (IPTG) at 22 °C for overnight. The cells were collected and re-suspended in GST buffer (25 mM Tris pH 8.0, 5 mM dithiothreitol (DTT), 150 mM NaCl) and sonicated (4 cycles/5 s each). After centrifugation, the supernatant was incubated with glutathione Sepharose beads for 1 h at 4 °C and subsequently washed four times before use in mTORC1 kinase assay.

### 4.5 mTORC1 *in vitro* kinase assay

The *in vitro* mTORC1 kinase assay was performed as previously described with a minor modification [11]. Briefly, HEK-293 cells stably expressing a control vector or Minar2 were serum-starved for overnight followed with insulin (100 ng/mL) stimulation for 15 min. Cells were lysed in CHAPS lysis buffer (40 mM HEPES (pH7.4), 2 mM EDTA and 0.3% CHAPS) plus protease and phosphatase inhibitors. Whole cell lysates were subjected for immunoprecipitation by incubating the cell lysates with raptor antibody (2 μg) for 2–3 h at 4 °C followed by an incubation of 1hr with Protein A/G Sepharose beads (GE Healthcare). The immunoprecipitates were washed (3X) with the CHAPS lysis buffer followed by an additional wash with a buffer containing 25 mM HEPES (pH7.4) and 20 mM KCl. *In vitro* kinase assay was carried out by incubating the immunoprecipitates with approximately 0.5 μg of E. coli purified GST-4E-BP1 as the substrate for mTORC1 and incubated for 20 min at 30 °C in the mTORC1 kinase buffer (25 mM HEPES (pH7.4), 50 mM KCl, 10 mM MgCl2 and 250 μM ATP). The kinase reaction was stopped by the addition of 2X SDS sample buffer followed by incubation at 95 °C for 5 min. The samples were analyzed by SDS–PAGE and immunoblotted with a phospho-Thr37/46 4EBP1 antibody.

### 4.6 Real-time RT-PCR analysis

Total RNA was extracted using the RNeasy mini kit (Qiagen) and the reverse transcription reaction was performed using the reverse transcription supermix (Bio-Rad) and real-time RT–PCR analysis was performed using the SYBR Green PCR master mix (ABI, 4,367,659). The primers used was previously described [17].

Pparg, Fabp4 and adiponectin are markers for mature adipocytes.

Pparg, F: GTGCCAGTTTCGATCCGTAGA,

Pparg, R: GGCCAGCATCGTGTAGATGA;

Adiponectin, F: GCACTGGCAAGTTCTACTGCAA,

Adiponectin, R: GTAGGTGAAGAGAACGGCCTTGT;

Fabp4, F: GAATTCGATGAAATCACCGCA, Fabp4, R:CTCTTTATTGTGGTCGACTTTCCA;

Hprt, F: AGCCTAAGATGAGCGCAAGT, Hprt, R: TTACTAGGCAGATGGCCACA. The mRNA level of Hprt was used as an internal control.

### 4.7 High-fat diet-induced obesity model

Six-week-old female and male *Minar2* KO mice and their WT littermates were fed with a standard chow diet (4.5% fat, Labdiet) or a high-fat diet (HFD) (35% fat, Research diets, Cat #D12492) and bodyweight was weekly monitored up to 7 weeks. Food intake of different groups was monitored for at least four weeks. At the end of the experiment, mice were euthanized, key organs/tissues and blood samples were collected for further analysis.

### 4.8 Histological analyses

Mouse tissues were harvested and fixed with 4% paraformaldehyde (PFA), dehydrated, and embedded in paraffin. Paraffin sections (5 μm) were prepared and subjected to hematoxylin and Eosin (H&E) or other staining procedures.

### 4.9 Mass spectrometry analyses

Mass spectrometry analyses of HEK-293 cells expressing Minar2 immunoprecipitated proteins was carried out as described previously [2,16]. Briefly, nUPLC-MS/MS analyses were performed on an Orbitrap Fusion Lumos Tribrid mass spectrometer (ThermoFisher Scientific, Waltham, MA) coupled with an ACQUITY UPLC M-Class system (Waters Corp., Milford, MA) and a TriVersa NanoMate (Advion, Ithaca, NY). For LC separation, a nanoEase Symmetry C18 UPLC Trap Column (100 Å, 5 μm, 180 μm × 20 mm, Waters) was used as the trapping column, and a nanoEase MZ HSS C18 T^3^ UPLC Column (100 Å, 1.8 μm, 75 μm × 100 mm, Waters) was used as the analytical column. The peptides were trapped at 4 μL/min for 4 min with 1% acetonitrile and 0.1% formic acid (Solvent A). Following the trapping step, peptides were separated on the analytical column according to the following conditions: 0–1 min: 2% B, 1–3 min: 2–5% B, 3–43 min: 5–40% B (Solvent B: 99% acetonitrile and 0.1% formic acid). All mass spectrometry (MS) analyses were performed in the positive mode with the RF lens set to 30%. MS scans were acquired with the following settings: 120,000 resolution at *m*/*z* 200, scan range *m*/*z* 370–2000, 1 μscan/MS, Normalized AGC target 250%, and a maximum injection time of 50 ms. For HCD analyses, MS2 scans (NCE 28%) were acquired with the following settings: 30,000 resolution at *m*/*z* 200, Mass range and Scan Range Mode were set to Normal and Auto, respectively; 1 μscan/MS, AGC target 50%, and a maximum injection time of 60 ms. MS/MS data were searched against 20,352 entires in an UniProtKB database restricted to *Homo sapiens* (downloaded in May, 2021) using the Andromeda search engine from MaxQuant v1.6.14. (Max Planck Inst. Biochem.). Carbamidomethylation (C) was set as fixed modification, whereas Met oxidation, protein N-terminal acetylation, and Phosphorylation at S/T/Y residue were defined as variable modifications. Mass tolerance was set to 10 and 20 ppm at the MS and MS/MS level. Enzyme specificity was set to trypsin with a maximum of two missed cleavages. Peptide-to-spectrum match False discovery rate was set as ≤ 1%.

### 4.10 MRI data acquisition

MRI experiments were performed on freshly sacrificed mice using a 9.4 T Bruker BioSpec system. Coronal T1-wighted images were acquired using a Rapid Imaging with Refocused Echoes (RARE) sequence to reveal hyperintensive signals from fat. Key parameters were: Repetition time (TR) = 800 ms, echo time (TE) = 6.68 ms, field of view (FOV) = 56 × 35 mm2, acquisition matrix = 384 × 256, slice thickness = 0.8 mm, slice gap = 0.2 mm, slice number = 22, number of average = 2.

### 4.11 Statistical analysis

Differences between two groups were applied for unpaired two-tailed Student's t-tests. All values are expressed as mean ± SD or as indicated. For all tests, ∗P < 0.05, ∗∗P < 0.01, and ∗∗∗P < 0.001 were considered statistically significant.

## Declaration of Competing Interest

The authors declare that they have no known competing financial interests or personal relationships that could have appeared to influence the work reported in this paper.

## Data Availability

No data was used for the research described in the article.
