# Supplementary material for: Inactivation of Minar2 in mice hyperactivates mTOR signaling and results in obesity
Source: Mol Metab. 2023 May 26;73:101744. doi: 10.1016/j.molmet.2023.101744 (PMC10267597; doi:10.1016/j.molmet.2023.101744)
Supplement: Multimedia component 1 [file mmc1.pdf]

Supplemental Information

Inactivation of *Minar2* in Mice Hyperactivates mTOR Signaling and Results in Obesity

Saran Lotfollahzadeh[1], Chaoshuang Xia[2], Razie Amraei[3], Ning Hua[4], Konstantin V. Kandror[5], Stephen R Farmer[5], Wenyi Wei[6], Catherine E Costello[2,5*], Vipul Chitalia[1,7,8*], Nader Rahimi[3*]

[1]Renal Section, Department of Medicine, Boston University Chobanian & Avedisian School of Medicine, Boston, MA, USA. [2]Center for Biomedical Mass Spectrometry, Boston University Chobanian & Avedisian School of Medicine, Boston. [3]Department of Pathology and Laboratory Medicine, Boston University Chobanian & Avedisian School of Medicine, Boston, MA, USA. [4]Biomed Research Center, Boston University Chobanian & Avedisian School of Medicine. [5]Department of Biochemistry, Boston University Chobanian & Avedisian School of Medicine, Boston, MA, USA. [6]Department of Pathology, Beth Israel Deaconess Medical Center, Harvard Medical School, Boston, MA, 02215 USA. [7]Veterans Affairs Boston Healthcare System, Boston MA, USA. [8]Institute of Medical Engineering and Sciences, Massachusetts Institute of Technology, Cambridge, MA,USA.

Key words: MINAR2, mTOR, mTORC1, RAPTOR, obesity

***Co-corresponding Authors:**
Nader Rahimi
Email: nrahimi@bu.edu
Catherine E Costello
Email: cecmsms@bu.edu
Vipul Chitalia
Email: vichital@bu.edu

Acknowledgment and Funding:
This work was supported in part through a grant from Boston University School of Medicine Genome Science Institute (to C.E.C. and N.R.), the Center of Cross-Organ Vascular Pathology, DOM, BU (VC) and NIH grants R24 GM134210, S10 OD021728, S10 OD010724 (C.E.C.).

S. Figure 1. **MRI analysis shows *Minar2* KO mice have increase fat mass.** (**A**) MRI analysis of Minar2 KO and sex- and age-matched control WT mice. F, female, M, Male. (**B**) The graph is the quantification of adipose tissue (6 weeks old, n=4/group, 2 males and 2 females/group) of MRI analysis.

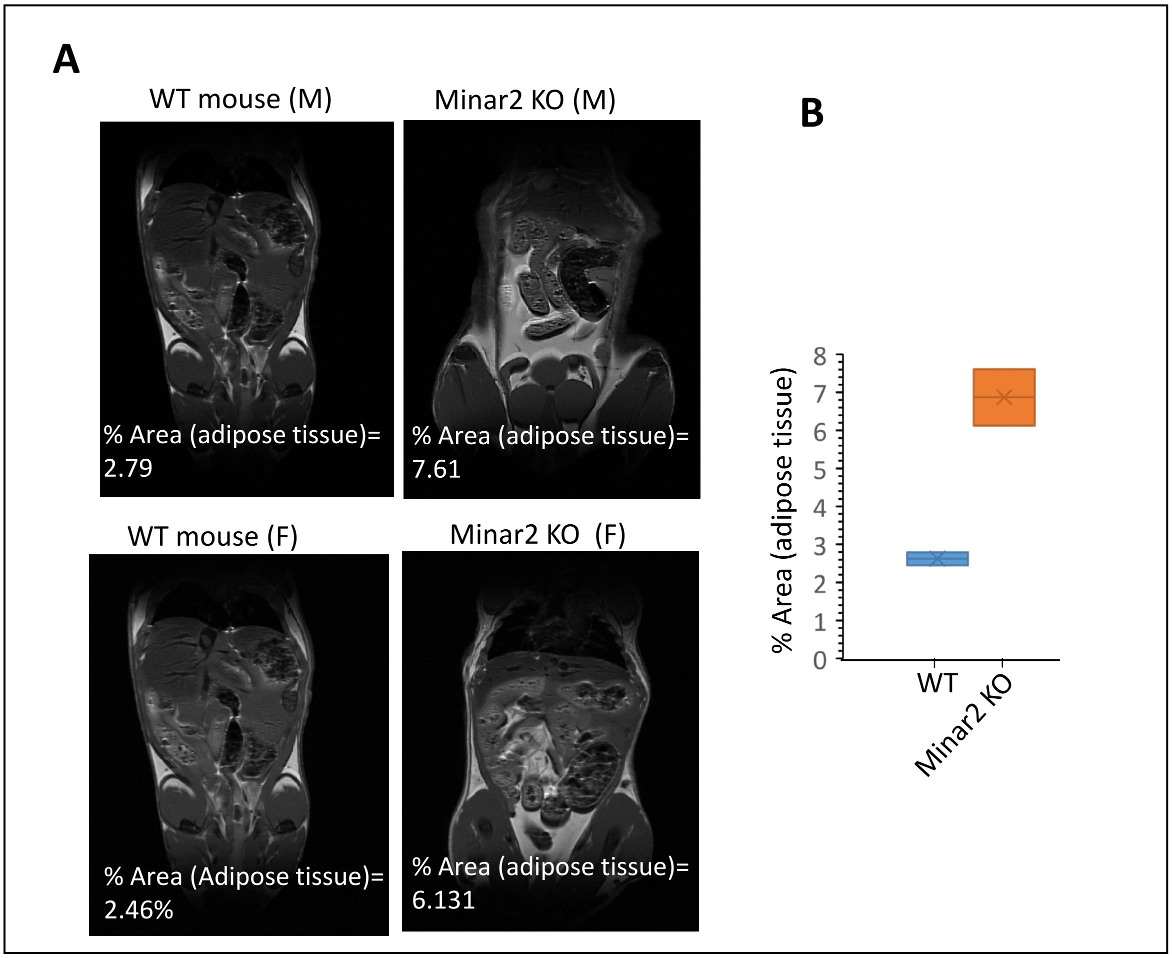

S. Figure 2. **Adipocyte cell numbers are not altered in Minar2 KO mice**. The number of adipocytes from H&E slides were calculated by Image J software. The graph is representative of three randomly but non-overlapping areas per slide, mean ± SEM, *n*=3). *P*>0.05 is considered not significant.

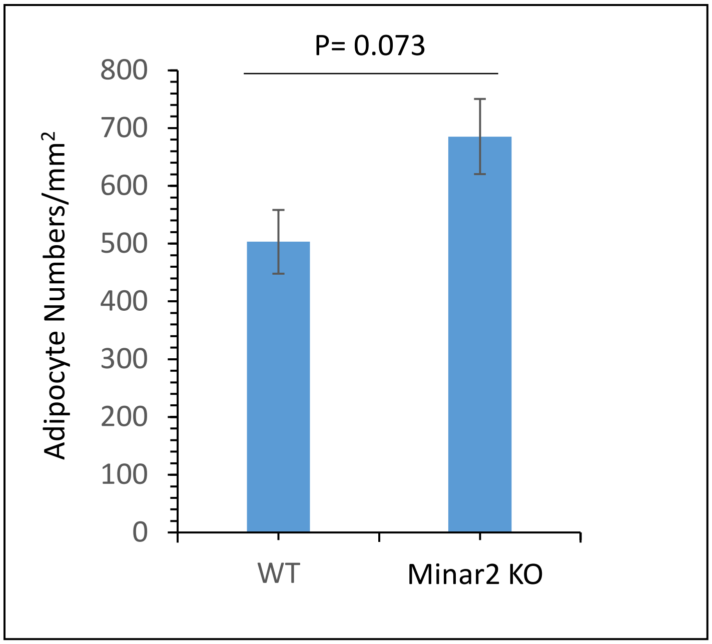

S. Figure 3. Raptor co-localizes with Minar2. (**A**) HEK-293 cells expressing GST-Raptor alone or GST-Raptor with Minar2-emarald (EM) were stained anti-GST antibody. Image magnification 50 μM. (**B**) HEK-293 cells or HEK-293 cells expressing GST-Raptor with calreticulin (CAL) mCherry. Image magnification 50 μM. (**C**) HEK-293 cells or HEK-293 cells co-expressing GST-Raptor and Lysosome20-emarald (EM). Image magnification 50 μM.

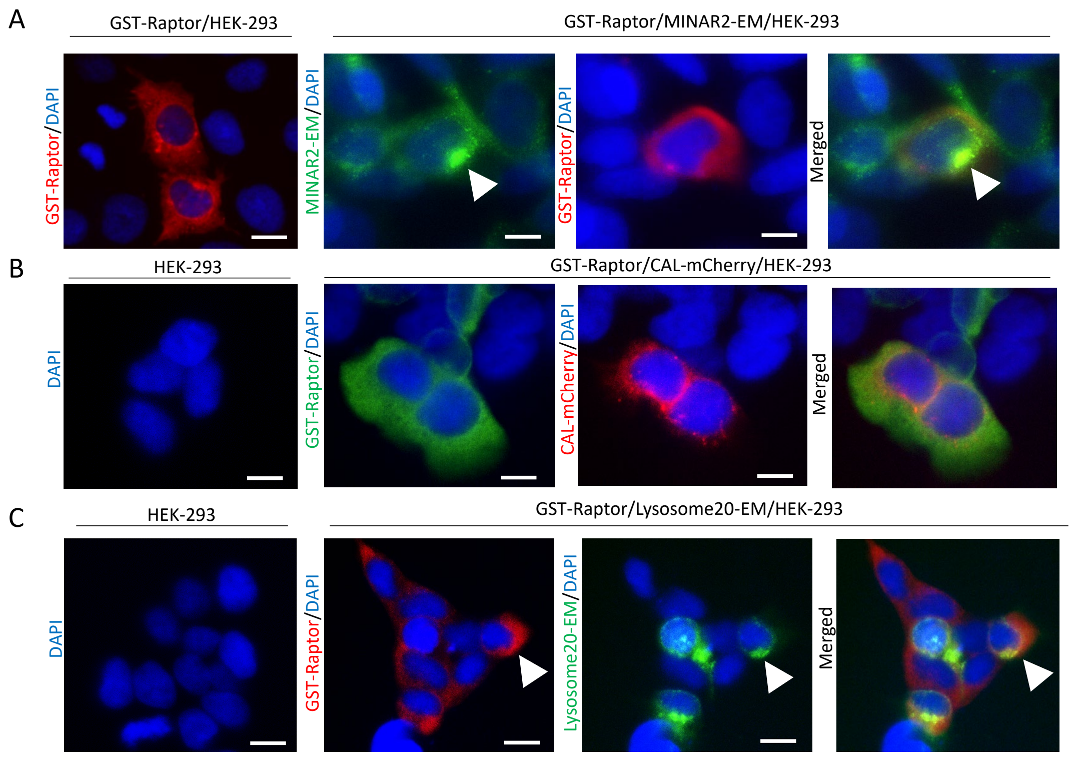

S. Figure 4. **Raptor is phosphorylated on Ser 863 and Ser877**. Higher energy collisional dissociation (HCD) tandem mass spectra of Raptor tryptic phosphor-peptides that contain the S863 and S877 phosphorylation sites. NanoUPLC retention times and fragmentation patterns were matched.

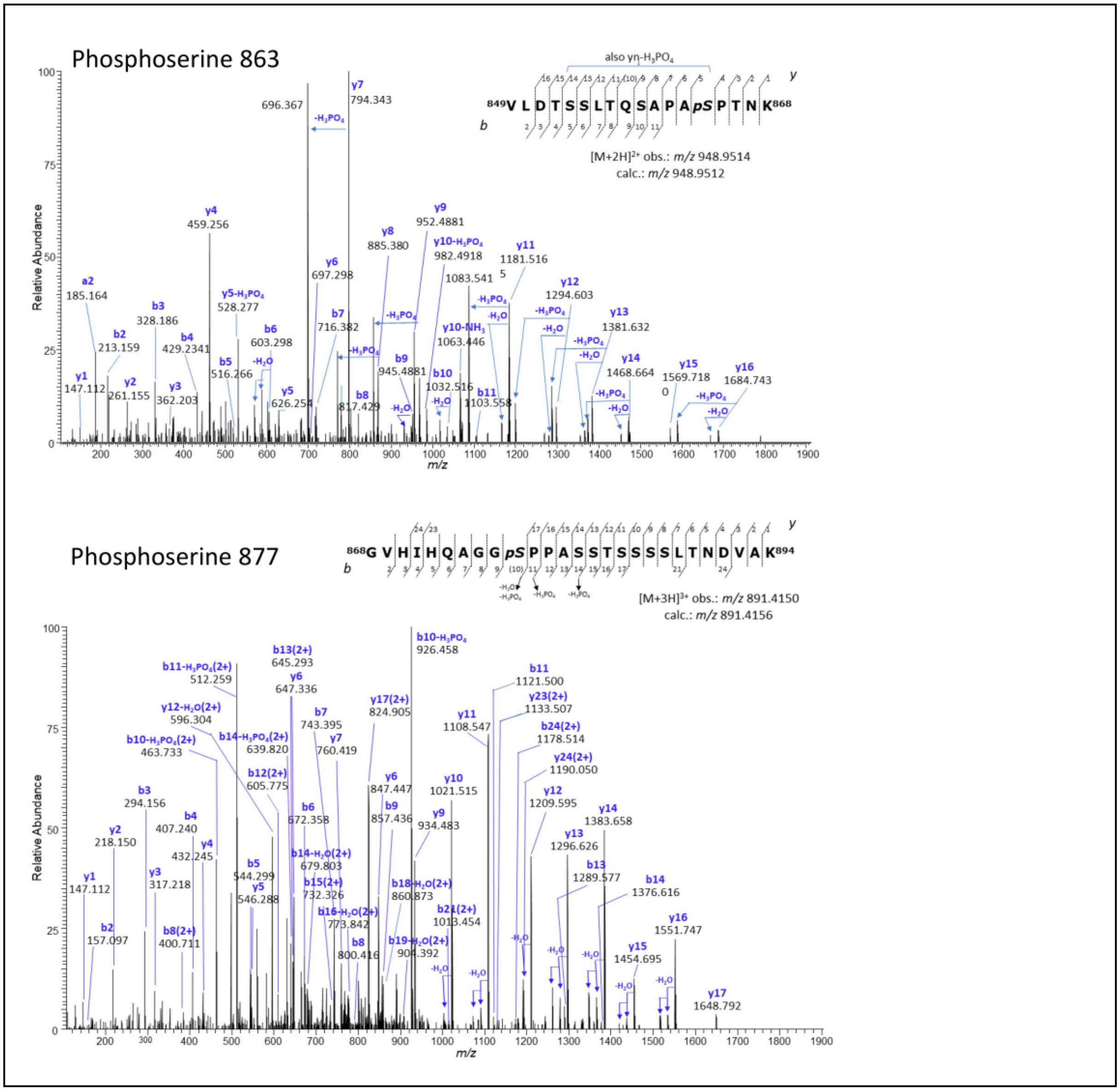

S. Figure 5. *Minar2* cDNA and vector sequence information. *Minar2* sequence is highlighted in yellow. Myc tag sequence is underlined. Emerald sequence (795bp) is highlighted in green.

>Minar2-pQCXIP

tccgcgttacataacttacggtaaatggcccgcctggctgaccgcccaacgacccccgcccattgacgtcaataatgacgtatgtt
cccatagtaacgccaatagggactttccattgacgtcaatgggtggagtatttacggtaaactgcccacttggcagtacatcaagg
tatcatatgccaagtacgccccctattgacgtcaatgacggtaaatggcccgcctggcattatgcccagtacatgaccttatggga
ctttcctacttggcagtacatctacgtattagtcatcgctattaccatggtgatgcggttttggcagtacatcaatgggcgtggat
agcggtttgactcacggggatttccaagtctccaccccattgacgtcaatgggagtttgttttggcaccaaaatcaacgggacttt
ccaaaatgtcgtaacaactccgccccattgacgcaaatgggcggtaggcgtgtacggtgggaggtctatataagcagagctcaata
aaagagcccacaacccctcactcggcgcgccagtcttccgatagactgcgtcgcccgggtacccgtattcccaataaagcctcttg
ctgtttgcatccgaatcgtggtctcgctgttccttgggagggtctcctctgagtgattgactacccacgacgggggtctttcattt
gggggctcgtccgggatttggagacccctgcccagggaccaccgacccaccaccgggaggtaagctggccagcaacttatctgtgt
ctgtccgattgtctagtgtctatgtttgatgttatgcgcctgcgtctgtactagttagctaactagctctgtatctggcggacccg
tggtggaactgacgagttctgaacacccggccgcaaccctgggagacgtcccagggactttgggggccgtttttgtggcccgacct
gaggaagggagtcgatgtggaatccgaccccgtcaggatatgtggttctggtaggagacgagaacctaaaacagttcccgcctccg
tctgaatttttgctttcggtttggaaccgaagccgcgcgtcttgtctgctgcagcgctgcagcatcgttctgtgttgtctctgtct
gtgtttctgtatttgtctgaaaattagggccagactgttaccactcccttaagtttgaccttaggtcactggaaagatgtcgagcg
atcgctcacaaccagtcggtagatgtcaagaagagacgttgggttaccttctgctctgcagaatggccaacctttaacgtcggatg
ccgcgagacggcacctttaaccgagacctcatcacccaggttaagatcaaggtcttttcacctggcccgcatggacacccagaccg
gtcccctacatcgtgacctgggaagccttggcttttgacccccctccctgggtcaagccctttgtacaccctaagcctccgcctcc
tcttcctccatccgccccgtctctcccccttgaacctcctcgttcgaccccgcctcgatcctccctttatccagccctcactcctt
ctctaggcgccggaattgaagatctgggggatcgatcctctagagtccgttacataacttacggtaaatggcccgcctggctgacc
gcccaacgacccccgcccattgacgtcaataatgacgtatgttcccatagtaacgccaatagggactttccattgacgtcaatggg
tggagtatttacggtaaactgcccacttggcagtacatcaagtgtatcatatgccaagtacgccccctattgacgtcaatgacggt
aaatggcccgcctggcattatgcccagtacatgaccttatgggactttcctacttggcagtacatctacgtattagtcatcgctat
taccatggtgatgcggttttggcagtacatcaatgggcgtgaatagcggtttgactcacggggatttccaagtctccaccccattg
acgtcaatgggagtttgttttggcaccaaaatcaacgggactttccaaaatgtcgtaacaactccgccccattgacgcaaatgggc
ggtaggcgtgtacgtgggaggtctatataagcagagctcgtttagtgaaccgtcagatcgcctggagacgccatccacgctgtttt
gacctccatagaagacaccgggaccgatccagcctccgcggccgggaacggtgcattggaacgctgcaggaattgatccgcggccg
c**ATG**GATCTCTCTGTTTTGCCAAATAACAACCATCCTGACAAATTCCTGCAGCTTGACGTAAAGTCTTTAACGAGGAGCTCAGCCC
TCCTTCAGGCCAGCCTGGTGAGGTTTCCGGGTGGAAATTATCCTGCTGCACAACACTGGCAAAACCTTGTCTACTCACAGAGGGAA
AAGAAGAATATTGCTGCTCAACGAATTAGGGGATCCAGTGCAGACAGCCTTGTCACTGCTGATAGCCCCCCACCATCCATGTCATC
AGTTATGAAGAATAACCCACTCTATGGTGACCTAAGTTTGGAGGAAGCTATGGAAGAAAGAAAAAAGAACCCCTCATGGACCATTG
AGGAATATGACAAACATTCCCTGCACACAAACCTCTCTGGACATCTGAAGGAAAATCCTAATGACCTGCGGTTTTGGTTGGGAGAC
ATGTACACTCCAGGTTTTGACACTTTATTGAAAAAGGAAGAGAAACAAGAGAAGCATTCAAAATTCTGTCGTATGGGTCTGATTTT
ACTTGTCGTTATCTCCATCTTGGTTACCATAGTGACTATCATTACTTTTTTCACCGGATCCAGGGGAACAAAAACTCATCTCAGAA
GAGGATCTG**TGA**AAATTTCgaattccgcccctctccctcccccccccctaacgttactggccgaagccgcttggaataagccggtg
tgcgtttgtctatatgttattttccaccatattgccgtcttttggcaatgtgagggcccggaaacctggccctgtcttcttgacga
gcattcctaggggtctttcccctctcgccaaaggaatgcaaggtctgttgaatgtcgtgaaggaagcagttcctctggaagcttct
tgaagacaaacaacgtctgtagcgaccctttgcaggcagcggaaccccccacctggcgacaggtgcctctgcggccaaaagccacg
tgtataagatacacctgcaaaggcggcacaaccccagtgccacgttgtgagttggatagttgtggaaagagtcaaatggctctcct
caagcgtattcaacaaggggctgaaggatgcccagaaggtaccccattgtatgggatctgatctggggcctcggtgcacatgcttt
acatgtgtttagtcgaggttaaaaaacgtctaggccccccgaaccacggggacgtggttttcctttgaaaaacacgatgataagct
tgccacaacccacaaggagacgaccttccatgaccgagtacaagcccacggtgcgcctcgccacccgcgacgacgtcccccgggcc
gtacgcaccctcgccgccgcgttcgccgactaccccgccacgcgccacaccgtcgacccggaccgccacatcgagcgggtcaccga
gctgcaagaactcttcctcacgcgcgtcgggctcgacatcggcaaggtgtgggtcgcggacgacggcgccgcggtggcggggacca
cgccggagagcgtcgaagcgggggcggtgttcgccgagatcggcccgcgcatggccgagttgagcggttcccggctggccgcgcag
caacagatggaaggcctcctggcgccgcaccggcccaaggagcccgcgtggttcctggccaccgtcggcgtctcgcccgaccagg
gcaagggtctgggcagcgccgtcgtgctccccggagtggaggcggccgagcgcgccggggtgcccgccttcctggagacctccgcg
ccccgcaacctccccttctacgagcggctcggcttcaccgtcaccgccgacgtcgagtgcccgaaggaccgcgcgacctgtgcatg
acccgcaagcccggtgcctgacgcccgccccacgacccgcagcgcccgaccgaaaggagcgcacgaccccatggctccgaccgaag
ccgacccgggcggccccgccgaccccgcacccgcccccgaggcccaccgactctagtcgagggctgcagcgctgcagaggccgagt
gcagaactgctccaaaggacctcaaggctttccgagggacactaggctgactccatcgagccagtgtagagataagcttatcgat
tagtccaatttgttaaagacaggatatcagtggtccaggctctagttttgactcaacaatatcaccagctgaagcctatagagtac
gagccatagataaaataaaagattttatttagtctccagaaaaaggggggaatgaaagaccccacctgtaggtttggcaagctagc
ttaagtaacgccattttgcaaggcatggaaaaatacataactgagaatagagaagttcagatcaaggtcaggaacagatggaacag
ggtcgaccctagagaaccatcagatgtttccagggtgccccaaggacctgaaatgaccctgtgccttatttgaactaaccaatcag
ttcgcttctcgcttctgttcgcgcgcttctgctccccgagctcaataaaagagcccacaacccctcactcggggcgccagtcctcc
gattgactgagtcgcccgggtacccgtgtatccaataaaccctcttgcagttgcatccgacttgtggtctcgctgttccttgggag
ggtctcctctgagtgattgactacccgtcagcgggggtctttcatttgggggctcgtccgggatcgggagacccctgcccagggac
caccgacccaccaccgggaggtaagctggctgcctcgcgcgtttcggtgatgacggtgaaaacctctgacacatgcagctcccgga

gacggtcacagcttgtctgtaagcggatgccgggagcagacaagcccgtcagggcgcgtcagcgggtgttggcgggtgtcggggcg
cagccatgacccagtcacgtagcgatagcggagtgtagatccggctgtggaatgtgtgtcagttagggtgtggaaagtccccaggc
tccccagcaggcagaagtatgcaaagcatgcatctcaattagtcagcaaccaggtgtggaaagtccccaggctccccagcaggcag
aagtatgcaaagcatgcatctcaattagtcagcaaccatagtcccgcccctaactccgcccatcccgcccctaactccgcccagtt
ccgcccattctccgccccatggctgactaattttttttatttatgcagaggccgaggccgcctcggcctctgagctattccagaag
tagtgaggaggcttttttggaggcctaggcttttgcaaaaagcttactggcttaactatgcggcatcagagcagattgtactgaga
gtgcaccatatgcggtgtgaaataccgcacagatgcgtaaggagaaaataccgcatcaggcgctcttccgcttcctcgctcactga
ctcgctgcgctcggtcgttcggctgcggcgagcggtatcagctcactcaaaggcggtaatacggttatccacagaatcaggggata
acgcaggaaagaacatgtgagcaaaaggccagcaaaaggccaggaaccgtaaaaaggccgcgttgctggcgtttttccataggctc
cgcccccctgacgagcatcacaaaaatcgacgctcaagtcagaggtggcgaaacccgacaggactataaagataccaggcgtttcc
ccctggaagctccctcgtgcgctctcctgttccgaccctgccgcttaccggatacctgtccgcctttctcccttcgggaagcgtgg
cgctttctcatagctcacgctgtaggtatctcagttcggtgtaggtcgttcgctccaagctgggctgtgtgcacgaaccccccgtt
cagcccgaccgctgcgccttatccggtaactatcgtcttgagtccaacccggtaagacacgacttatcgccactggcagcagccag
taacaggattagcagagcgaggtatgtaggcggtgctacagagttcttgaagtggtggcctaactacggctacactagaaggacag
tatttggtatctgcgctctgctgaagccagttaccttcggaaaaagagttggtagctcttgatccggcaaacaaaccaccgctggt
agcggtggtttttttgtttgcaagcagcagattacgcgcagaaaaaaaggatctcaagaagatcctttgatcttttctacggggtc
tgacgctcagtggaacgaaaactcacgttaagggattttggtcatgagattatcaaaaaggatcttcacctagatccttttaaatt
aaaaatgaagttttaaatcaatctaaagtatatatgagtaaacttggtctgacagttaccaatgcttaatcagtgaggcacctatc
tcagcgatctgtctatttcgttcatccatagttgcctgactccccgtcgtgtagataactacgatacgggagggcttaccatctgg
ccccagtgctgcaatgataccgcgagacccacgctcaccggctccagatttatcagcaataaaccagccagccggaagggccgagc
gcagaagtggtcctgcaactttatccgcctccatccagtctattaattgttgccgggaagctagagtaagtagttcgccagttaat
agtttgcgcaacgttgttgccattgctgcaggcatcgtggtgtcacgctcgtcgtttggtatggcttcattcagctccggttccca
acgatcaaggcgagttacatgatcccccatgttgtgcaaaaaagcggttagctccttcggtcctccgatcgttgtcagaagtaagt
tggccgcagtgttatcactcatggttatggcagcactgcataattctcttactgtcatgccatccgtaagatgcttttctgtgacg
gtgagtactcaaccaagtcattctgagaatagtgtatgcggcgaccgagttgctcttgcccggcgtcaacacgggataataccgcc
cacatagcagaactttaaaagtgctcatcattggaaaacgttcttcggggcgaaaactctcaaggatcttaccgctgttgagatcg
ttcgatgtaacccactcgtgcacccaactgatcttcagcatcttttactttcaccagcgtttctgggtgagcaaaaacaggaaggc
aaaatgccgcaaaaaagggaataagggcgacacggaaatgttgaatactcatactcttcctttttcaatattattgaagcatttat
cagggttattgtctcatgagcggatacatatttgaatgtatttagaaaaataaacaaataggggttccgcgcacatttccccgaat
gccacctgacgtctaagaaaccattattatcatgacattaacctataaaaataggcgtatcacgaggccctttcgtcttcaagaat
tagcttggccattgcatacgttgtatccatatcataatatgtacatttatattggctcatgtccaacattaccgccatgttgacat
tgattattgactagttattaatagtaatcaattacggggtcattagttcatagcccatatatggagt

>Minar2-emerald-pLNCX

TTTGAAAGACCCCACCCGTAGGTGGCAAGCTAGCTTAAGTAACGCCACTTTGCAAGGCATGGAAAAATACATAACTGAGAATAGAA
AAGTTCAGATCAAGGTCAGGAACAAAGAAACAGCTGAATACCAAACAGGATATCTGTGGTAAGCGGTTCCTGCCCCGGCTCAGGGC
CAAGAACAGATGAGACAGCTGAGTGATGGGCCAAACAGGATATCTGTGGTAAGCAGTTCCTGCCCCGGCTCGGGGCCAAGAACAGA
TGGTCCCCAGATGCGGTCCAGCCCTCAGCAGTTTCTAGTGAATCATCAGATGTTTCCAGGGTGCCCCAAGGACCTGAAAATGACCT
GTACCTTATTTGAACTAACCAATCAGTTCGCTTCTCGCTTCTGTTCGCGCGCTTCCGCTCTCCGAGCTCAATAAAAGAGCCCACAA
CCCCTCACTCGGCGCGCCAGTCTTCCGATAGACTGCGTCGCCCGGGTACCCGTATTCCCAATAAAGCCTCTTGCTGTTTGCATCCG
AATCGTGGTCTCGCTGTTCCTTGGGAGGGTCTCCTCTGAGTGATTGACTACCCACGACGGGGGTCTTTCATTTGGGGGCTCGTCCG
GGATTTGGAGACCCCTGCCCAGGGACCACCGACCCACCACCGGGAGGTAAGCTGGCCAGCAACTTATCTGTGTCTGTCCGATTGTC
TAGTGTCTATGTTTGATGTTATGCGCCTGCGTCTGTACTAGTTAGCTAACTAGCTCTGTATCTGGCGGACCCGTGGTGGAACTGAC
GAGTTCTGAACACCCGGCCGCAACCCTGGGAGACGTCCCAGGGACTTTGGGGGCCGTTTTTGTGGCCCGACCTGAGGAAGGGAGTC
GATGTGGAATCCGACCCCGTCAGGATATGTGGTTCTGGTAGGAGACGAGAACCTAAAACAGTTCCCGCCTCCGTCTGAATTTTTGC
TTTCGGTTTGGAACCGAAGCCGCGCGTCTTGTCTGCTGCAGCGCTGCAGCATCGTTCTGTGTTGTCTCTGTCTGACTGTGTTTCTG
TATTTGTCTGAAAATTAGGGCCAGACTGTTACCACTCCCTTAAGTTTGACCTTAGGTCACTGGAAAGATGTCGAGCGGATCGCTCC
AACCAGTCGGTAGATGTCAAGAAGAGACGTTGGGTTACCTTCTGCTCTGCAGAATGGCCAACCTTTAACGTCGGATGGCCGCGAGC
GGCACCTTTAACCGAGACCTCATCACCCAGGTTAAGATCAAGGTCTTTTCACCTGGCCCGCATGGACACCCAGACCAGGTCCCCTA
CATCGTGACCTGGGAAGCCTTGGCTTTTGACCCCCCTCCCTGGGTCAAGCCCTTTGTACACCCTAAGCCTCCGCCTCCTCTTCCTC
ATCCGCCCCGTCTCTCCCCCTTGAACCTCCTCGTTCGACCCCGCCTCGATCCTCCCTTTATCCAGCCCTCACTCCTTCTCTAGGCG
CCGGAATTCCGATCTGATCAAGAGACAGGATGAGGATCGTTTCGCATGATTGAACAAGATGGATTGCACGCAGGTTCTCCGGCCGC
TTGGGTGGAGAGGCTATTCGGCTATGACTGGGCACAACAGACAATCGGCTGCTCTGATGCCGCCGTGTTCCGGCTGTCAGCGCAGG
GGCGCCCGGTTCTTTTTGTCAAGACCGACCTGTCCGGTGCCCTGAATGAACTGCAGGACGAGGCAGCGCGGCTATCGTGGCTGGCC
ACGACGGGCGTTCCTTGCGCAGCTGTGCTCGACGTTGTCACTGAAGCGGGAAGGGACTGGCTGCTATTGGGCGAAGTGCCGGGGCA
GGATCTCCTGTCATCTCACCTTGCTCCTGCCGAGAAAGTATCCATCATGGCTGATGCAATGCGGCGGCTGCATACGCTTGATCCGG
CTACCTGCCCATTCGACCACCAAGCGAAACATCGCATCGAGCGAGCACGTACTCGGATGGAAGCCGGTCTTGTCGATCAGGATGAT
CTGGACGAAGAGCATCAGGGGCTCGCGCCAGCCGAACTGTTCGCCAGGCTCAAGGCGCGCATGCCCGACGGCGAGGATCTCGTCGT
GACCCATGGCGATGCCTGCTTGCCGAATATCATGGTGGAAAATGGCCGCTTTTCTGGATTCATCGACTGTGGCCGGCTGGGTGTGG
CGGACCGCTATCAGGACATAGCGTTGGCTACCCGTGATATTGCTGAAGAGCTTGGCGGCGAATGGGCTGACCGCTTCCTCGTGCTT
TACGGTATCGCCGCTCCCGATTCGCAGCGCATCGCCTTCTATCGCCTTCTTGACGAGTTCTTCTGAGCGGGACTCTGGGGTTCGAA

ATGACCGACCAAGCGACGCCCAACCTGCCATCACGAGATTTCGATTCCACCGCCGCCTTCTATGAAAGGTTGGGCTTCGGAATCGT
TTTCCGGGACGCCGGCTGGATGATCCTCCAGCGCGGGGATCTCATGCTGGAGTTCTTCGCCCACCCCGGGCTCGATCCCCTCGCGA
GTTGGTTCAGCTGCTGCCTGAGGCTGGACGACCTCGCGGAGTTCTACCGGCAGTGCAAATCCGTCGGCATCCAGGAAACCAGCAGC
GGCTATCCGCGCATCCATGCCCCCGAACTGCAGGAGTGGGGAGGCACGATGGCCGCTTTGGTCGAGGCGGATCCGGCCATTAGCCA
TATTATTCATTGGTTATATAGCATAAATCAATATTGGCTATTGGCCATTGCATACGTTGTATCCATATCATAATATGTACATTTAT
ATTGGCTCATGTCCAACATTACCGCCATGTTGACATTGATTATTGACTAGTTATTAATAGTAATCAATTACGGGGTCATTAGTTCA
TAGCCCATATATGGAGTTCCGCGTTACATAACTTACGGTAAATGGCCCGCCTGGCTGACCGCCCAACGACCCCCGCCCATTGACGT
CAATAATGACGTATGTTCCCATAGTAACGCCAATAGGGACTTTCCATTGACGTCAATGGGTGGAGTATTTACGGTAAACTGCCCAC
TTGGCAGTACATCAAGTGTATCATATGCCAAGTACGCCCCCTATTGACGTCAATGACGGTAAATGGCCCGCCTGGCATTATGCCCA
GTACATGACCTTATGGGACTTTCCTACTTGGCAGTACATCTACGTATTAGTCATCGCTATTACCATGGTGATGCGGTTTTGGCAGT
ACATCAATGGGCGTGGATAGCGGTTTGACTCACGGGGATTTCCAAGTCTCCACCCCATTGACGTCAATGGGAGTTTGTTTTGGCAC
CAAAATCAACGGGACTTTCCAAAATGTCGTAACAACTCCGCCCCATTGACGCAAATGGGCGGTAGGCATGTACGGTGGGAGGTCTA
TATAAGCAGAGCTCGTTTAGTGAACCGTCAGATCGCCTGGAGACGCCATCCACGCTGTTTTGACCTCCATAGAAGACACCGGGACG
ATCCAGCCTCCGCGGCCCCGTTAACGCGGCCGC**ATG**GATCTCTCTGTTTTGCCAAATAACAACCATCCTGACAAATTCCTGCAGCT
TGACGTAAAGTCTTTAACGAGGAGCTCAGCCCTCCTTCAGGCCAGCCTGGTGAGGTTTCCGGGTGGAAATTATCCTGCTGCACAAC
ACTGGCAAAACCTTGTCTACTCACAGAGGGAAAAGAAGAATATTGCTGCTCAACGAATTAGGGGATCCAGTGCAGACAGCCTTGTC
ACTGCTGATAGCCCCCCACCATCCATGTCATCAGTTATGAAGAATAACCCACTCTATGGTGACCTAAGTTTGGAGGAAGCTATGGA
AGAAAGAAAAAAGAACCCCTCATGGACCATTGAGGAATATGACAAACATTCCCTGCACACAAACCTCTCTGGACATCTGAAGGAAA
ATCCTAATGACCTGCGGTTTTGGTTGGGAGACATGTACACTCCAGGTTTTGACACTTTATTGAAAAAGGAAGAGAAACAAGAGAAG
CATTCAAAATTCTGTCGTATGGGTCTGATTTTACTTGTCGTTATCTCCATCTTGGTTACCATAGTGACTATCATTACTTTTTTCAC
CAAGCTTGCATCCGGCGGTAGCGGCGGTGGCAGCGGACCGGTCGCCACCATGGTGAGCAAGGGCGAGGAGCTGTTCACCGGGGTGG
TGCCCATCCTGGTCGAGCTGGACGGCGACGTAAACGGCCACAAGTTCAGCGTGTCCGGCGAGGGCGAGGGCGATGCCACCTACGGC
AAGCTGACCCTGAAGTTCATCTGCACCACCGGCAAGCTGCCCGTGCCCTGGCCCACCCTCGTGACCACCTTGACCTACGGCGTGCA
GTGCTTCGCCCGCTACCCCGACCACATGAAGCAGCACGACTTCTTCAAGTCCGCCATGCCCGAAGGCTACGTCCAGGAGCGCACCA
TCTTCTTCAAGGACGACGGCAACTACAAGACCCGCGCCGAGGTGAAGTTCGAGGGCGACACCCTGGTGAACCGCATCGAGCTGAAG
GGCATCGACTTCAAGGAGGACGGCAACATCCTGGGGCACAAGCTGGAGTACAACTACAACAGCCACAAGGTCTATATCACCGCCGA
CAAGCAGAAGAACGGCATCAAGGTGAACTTCAAGACCCGCCACAACATCGAGGACGGCAGCGTGCAGCTCGCCGACCACTACCAGC
AGAACACCCCCATCGGCGACGGCCCCGTGCTGCTGCCCGACAACCACTACCTGAGCACCCAGTCCAAGCTGAGCAAAGACCCCAAC
GAGAAGCGCGATCACATGGTCCTGCTGGAGTTCGTGACCGCCGCCGGGATCACTCTCGGCATGGACGAGCTGTACAAGTAAGCGGC
CGCGACTCTAGATCATAATCAGCCAT**TGA**GTCGACAAAATAAAAGATTTTATTTAGTCTCCAGAAAAAGGGGGGAATGAAAGACCC
ACCTGTAGGTTTGGCAAGCTAGCTTAAGTAACGCCATTTTGCAAGGCATGGAAAAATACATAACTGAGAATAGAGAAGTTCAGATC
AAGGTCAGGAACAGATGGAACAGCTGAATATGGGCCAAACAGGATATCTGTGGTAAGCAGTTCCTGCCCCGGCTCAGGGCCAAGAA
CAGATGGAACAGCTGAATATGGGCCAAACAGGATATCTGTGGTAAGCAGTTCCTGCCCCGGCTCAGGGCCAAGAACAGATGGTCCC
CAGATGCGGTCCAGCCCTCAGCAGTTTCTAGAGAACCATCAGATGTTTCCAGGGTGCCCCAAGGACCTGAAATGACCCTGTGCCTT
ATTTGAACTAACCAATCAGTTCGCTTCTCGCTTCTGTTCGCGCGCTTCTGCTCCCCGAGCTCAATAAAAGAGCCCACAACCCCTCA
CTCGGGGCGCCAGTCCTCCGATTGACTGAGTCGCCCGGGTACCCGTGTATCCAATAAACCCTCTTGCAGTTGCATCCGACTTGTGG
TCTCGCTGTTCCTTGGGAGGGTCTCCTCTGAGTGATTGACTACCCGTCAGCGGGGGTCTTTCATTTGGGGGCTCGTCCGGGATCGG
GAGACCCCTGCCCAGGGACCACCGACCCACCACCGGGAGGTAAGCTGGCTGCCTCGCGCGTTTCGGTGATGACGGTGAAAACCTCT
GACACATGCAGCTCCCGGAGACGGTCACAGCTTGTCTGTAAGCGGATGCCGGGAGCAGACAAGCCCGTCAGGGCGCGTCAGCGGGT
GTTGGCGGGTGTCGGGGCGCAGCCATGACCCAGTCACGTAGCGATAGCGGAGTGTATACTGGCTTAACTATGCGGCATCAGAGAGA
TTGTACTGAGAGTGCACCATATGCGGTGTGAAATACCGCACAGATGCGTAAGGAGAAAATACCGCATCAGGCGCTCTTCCGCTTCG
CTCACTGACTCGCTGCGCTCGGTCGTTCGGCTGCGGCGAGCGGTATCAGCTCACTCAAAGGCGGTAATACGGTTATCCACAGAATC
AGGGGATAACGCAGGAAAGAACATGTGAGCAAAAGGCCAGCAAAAGGCCAGGAACCGTAAAAAGGCCGCGTTGCTGGCGTTTTCCA
TAGGCTCCGCCCCCCTGACGAGCATCACAAAAATCGACGCTCAAGTCAGAGGTGGCGAAACCCGACAGGACTATAAAGATACCAGG
CGTTTCCCCCTGGAAGCTCCCTCGTGCGCTCTCCTGTTCCGACCCTGCCGCTTACCGGATACCTGTCCGCCTTTCTCCCTTCGGGA
AGCGTGGCGCTTTCTCATAGCTCACGCTGTAGGTATCTCAGTTCGGTGTAGGTCGTTCGCTCCAAGCTGGGCTGTGTGCACGAACC
CCCCGTTCAGCCCGACCGCTGCGCCTTATCCGGTAACTATCGTCTTGAGTCCAACCCGGTAAGACACGACTTATCGCCACTGGCAG
CAGCCACTGGTAACAGGATTAGCAGAGCGAGGTATGTAGGCGGTGCTACAGAGTTCTTGAAGTGGTGGCCTAACTACGGCTACACT
AGAAGGACAGTATTTGGTATCTGCGCTCTGCTGAAGCCAGTTACCTTCGGAAAAAGAGTTGGTAGCTCTTGATCCGGCAAACAAAC
CACCGCTGGTAGCGGTGGTTTTTTTGTTTGCAAGCAGCAGATTACGCGCAGAAAAAAAGGATCTCAAGAAGATCCTTTGATCTTTT
CTACGGGGTCTGACGCTCAGTGGAACGAAAACTCACGTTAAGGGATTTTGGTCATGAGATTATCAAAAAGGATCTTCACCTAGATC
CTTTTAAATTAAAAATGAAGTTTTAAATCAATCTAAAGTATATATGAGTAAACTTGGTCTGACAGTTACCAATGCTTAATCAGTGA
GGCACCTATCTCAGCGATCTGTCTATTTCGTTCATCCATAGTTGCCTGACTCCCCGTCGTGTAGATAACTACGATACGGGAGGGCT
TACCATCTGGCCCCAGTGCTGCAATGATACCGCGAGACCCACGCTCACCGGCTCCAGATTTATCAGCAATAAACCAGCCAGCCGAA
GGGCCGAGCGCAGAAGTGGTCCTGCAACTTTATCCGCCTCCATCCAGTCTATTAATTGTTGCCGGGAAGCTAGAGTAAGTAGTCGC
CAGTTAATAGTTTGCGCAACGTTGTTGCCATTGCTGCAGGCATCGTGGTGTCACGCTCGTCGTTTGGTATGGCTTCATTCAGCTCC
GGTTCCCAACGATCAAGGCGAGTTACATGATCCCCCATGTTGTGCAAAAAAGCGGTTAGCTCCTTCGGTCCTCCGATCGTTGTAGA
AGTAAGTTGGCCGCAGTGTTATCACTCATGGTTATGGCAGCACTGCATAATTCTCTTACTGTCATGCCATCCGTAAGATGCTTTCT
GTGACTGGTGAGTACTCAACCAAGTCATTCTGAGAATAGTGTATGCGGCGACCGAGTTGCTCTTGCCCGGCGTCAACACGGGATAA
TACCGCGCCACATAGCAGAACTTTAAAAGTGCTCATCATTGGAAAACGTTCTTCGGGGCGAAAACTCTCAAGGATCTTACCGCTGT
TGAGATCCAGTTCGATGTAACCCACTCGTGCACCCAACTGATCTTCAGCATCTTTTACTTTCACCAGCGTTTCTGGGTGAGCAAAA
ACAGGAAGGCAAAATGCCGCAAAAAAGGGAATAAGGGCGACACGGAAATGTTGAATACTCATACTCTTCCTTTTTCAATATTATTG
AAGCATTTATCAGGGTTATTGTCTCATGAGCGGATACATATTTGAATGTATTTAGAAAAATAAACAAATAGGGGTTCCGCGCACAT

TTCCCCGAAAAGTGCCACCTGACGTCTAAGAAACCATTATTATCATGACATTAACCTATAAAAATAGGCGTATCACGAGGCCCTTT
CGTCTTCAAGAATTCATACCAGATCACCGAAAACTGTCCTCCAAATGTGTCCCCCTCACACTCCCAAATTCGCGGGCTTCTGCCTC
TTAGACCACTCTACCCTATTCCCCACACTCACCGGAGCCAAAGCCGCGGCCCTTCCGTTTCTTTGCT
